# Splicing Factor SF3B2 Regulates Cardiomyocyte Calcium Handling Through Alternative Splicing of Cardiac Ion Channel Genes

**DOI:** 10.64898/2026.07.25.740735

**Published:** 2026-07-28

**Authors:** Sean Murphy, Hanwen Wang, Nadine Zureick, Misato Koakutsu, David Suh, Dong Ik Lee, Chulan Kwon

## Abstract

**Background:**

Alternative splicing is a critical determinant of protein diversity in the heart, where it drives the postnatal functional maturation of cardiomyocytes and specifies the ion-channel and calcium-handling isoforms required for mature contractile function; dysregulated splicing programs have in turn been implicated in cardiomyopathies and arrhythmias. However, the splicing regulators that control cardiomyocyte calcium handling remain largely unknown.

**Objective:**

We systematically screened 276 splicing factor genes in human induced pluripotent stem cell-derived cardiomyocytes (hiPSC-CMs) to identify regulators of calcium handling, and we selected SF3B2, a core U2 snRNP spliceosome component, for detailed follow-up based on complex-level enrichment and prior identification by Murphy et al. 2021 [1].

**Methods:**

A high-throughput siRNA screen targeting 276 splicing factor genes was performed in hiPSC-CMs using 384-well calcium transient imaging. SF3B2 knockdown was followed by deep bulk RNA-seq analysis (n = 3 per group) for differential gene expression with DESeq2 and alternative splicing quantification with rMATS. RNA immunoprecipitation sequencing (RIP-seq) using an epitope-tagged SF3B2 construct was performed to identify direct mRNA binding targets of SF3B2.

**Results:**

The screen identified multiple U2 snRNP components, including SF3A2, SF3B1, and SF3B4—as regulators of calcium transient duration. SF3B2 was previously identified as a regulator of contractility in this screen [1], and the enrichment of its complex partners above the significance threshold in the current CTD75 analysis supported its selection for follow-up characterization. SF3B2 knockdown resulted in 2,683 differentially expressed genes (adjusted *p* < 0.05), with downregulated genes enriched in cell cycle and DNA replication pathways. Alternative splicing analysis revealed significant changes across all five rMATS event types in 36 genes within the cardiac muscle cell action potential involved in contraction gene ontology term (GO:0086002), including calcium channel ( *CACNA1C* , *CACNA1D* , *CACNA2D1* ), potassium channel ( *KCNH2* , *KCNQ1* ), and sodium channel ( *SCN5A* ) genes, although the large number of affected genes is consistent with broad spliceosomal perturbation and a formal enrichment test would be needed to determine whether cardiac action potential genes are preferentially affected. Integration of RIP-seq data (597 SF3B2-enriched transcripts, FDR < 0.05) with splicing analysis identified *CACNA2D1,* which encodes an auxiliary subunit of L-type calcium channels, as both directly bound by SF3B2 and alternatively spliced upon knockdown.

**Conclusion:**

These findings identify SF3B2 as a regulator of cardiomyocyte calcium handling and suggest that SF3B2-dependent missplicing of *CACNA2D1* may link core spliceosome function to the splicing programs underlying cardiomyocyte functional maturation.

**Significance Statement:**

This study provides the first systematic functional screen of splicing factors in cardiomyocyte calcium handling and identifies SF3B2, a U2 snRNP subunit, as a regulator of cardiac ion channel splicing. Transcriptomic, splicing, and RNA binding data converge on *CACNA2D1* , identifying a route by which a core splicing factor shapes cardiac contractility, extending cardiac splicing regulation beyond the accessory RBPs studied to date.

## Full Text Availability

The license terms selected by the author(s) for this preprint version do not permit archiving in PMC. The full text is available from the preprint server.

